# Exercise and Doxorubicin Modify Markers of Iron Overload and Cardiolipin Deficiency in Cardiac Mitochondria

**DOI:** 10.3390/ijms24097689

**Published:** 2023-04-22

**Authors:** Ryan N. Montalvo, Franccesco P. Boeno, Imtiaz M. Dowllah, Cesar E. Jacintho Moritz, Branden L. Nguyen, Vivian Doerr, Matthew P. Bomkamp, Ashley J. Smuder

**Affiliations:** Department of Applied Physiology & Kinesiology, University of Florida, Gainesville, FL 32611, USA

**Keywords:** heart, cardiotoxicity, anthracycline, oxidative stress, subsarcolemmal, intermyofibrillar

## Abstract

Doxorubicin (DOX) is a chemotherapeutic agent highly effective at limiting cancer progression. Despite the efficacy of this anticancer drug, the clinical use of DOX is limited due to cardiotoxicity. The cardiac mitochondria are implicated as the primary target of DOX, resulting in inactivation of electron transport system complexes, oxidative stress, and iron overload. However, it is established that the cardiac mitochondrial subpopulations reveal differential responses to DOX exposure, with subsarcolemmal (SS) mitochondria demonstrating redox imbalance and the intermyofibrillar (IMF) mitochondria showing reduced respiration. In this regard, exercise training is an effective intervention to prevent DOX-induced cardiac dysfunction. Although it is clear that exercise confers mitochondrial protection, it is currently unknown if exercise training mitigates DOX cardiac mitochondrial toxicity by promoting beneficial adaptations to both the SS and IMF mitochondria. To test this, SS and IMF mitochondria were isolated from sedentary and exercise-preconditioned female Sprague Dawley rats exposed to acute DOX treatment. Our findings reveal a greater effect of exercise preconditioning on redox balance and iron handling in the SS mitochondria of DOX-treated rats compared to IMF, with rescue of cardiolipin synthase 1 expression in both subpopulations. These results demonstrate that exercise preconditioning improves mitochondrial homeostasis when combined with DOX treatment, and that the SS mitochondria display greater protection compared to the IMF mitochondria. These data provide important insights into the molecular mechanisms that are in part responsible for exercise-induced protection against DOX toxicity.

## 1. Introduction

Doxorubicin (DOX) is an anthracycline antibiotic used to treat a broad spectrum of cancers [1,2,3,4,5]. Despite its efficacy, the clinical use of DOX is limited due to deleterious cardiac side effects [6,7,8,9]. While the mechanisms responsible for DOX cardiotoxicity remain unclear, mitochondrial dysfunction is implicated as a major contributor [10,11,12,13,14]. Specifically, DOX accumulates within cardiac mitochondria due to its affinity for cardiolipin, a phospholipid located on the inner mitochondria membrane. Formation of the DOX-cardiolipin complex can alter mitochondrial structure and function by disrupting cytochrome c anchoring and oxidative phosphorylation, and by promoting reactive oxygen species (ROS) formation and apoptosis [7,9,14]. Additionally, both clinical and preclinical studies have demonstrated a key role for aberrant mitochondrial electron transport, metabolism, and dynamics in the development and progression of heart disease [13,15,16]. Therefore, further insight into the precise mechanisms of DOX-induced mitochondrial dysfunction is needed to establish therapeutic strategies.

Cardiac muscle contains two distinct populations of mitochondria which differ in their inherent properties [17,18,19]. Subsarcolemmal (SS) mitochondria are located just below the cell membrane and exhibit greater resistance to mitochondria permeability transition pore (mPTP) opening and oxidative stress, whereas the intermyofibrillar (IMF) mitochondria are located within the myofibrils and have greater enzyme activities and respiration [20,21,22,23,24]. Evaluation of spatial differences in response to DOX treatment has revealed greater effects on the IMF mitochondria compared to the SS fraction, with IMF exhibiting greater deficits in mitochondrial complex I respiration and increased susceptibility to apoptosis [25,26].

In addition to their distinct properties and response to damaging stress, SS and IMF mitochondria exhibit unique modifications in response to exercise training, which are cardioprotective against apoptotic stimuli and ischemia reperfusion injury [17,18,27]. Although significant work has been undertaken to demonstrate the ability of exercise training to improve cardiac and mitochondrial function following DOX treatment, the precise adaptations of each subfraction are currently unknown. Thus, the purpose of this study was to elucidate the mitochondrial adaptive plasticity to both exercise and DOX treatment. To investigate the molecular determinants of exercise-mediated protection against DOX-induced mitochondriopathy and cardiotoxicity we independently examined the cardiac SS and IMF mitochondria subpopulations. Our results indicate that exercise preconditioning is a beneficial approach to improve cardiorespiratory capacity following DOX treatment, and that the cardiac mitochondrial subfractions are differentially affected by exercise and DOX treatment. These findings further the understanding of the unique effects of exercise and DOX on the cardiac mitochondria subpopulations, supporting exercise as a therapeutic and revealing potential pharmacological targets to prevent DOX toxicity.

## 2. Results

### 2.1. Exercise Preconditioning Limits Body Weight Loss and Exercise Intolerance in Response to DOX Treatment

**Figure 1:**
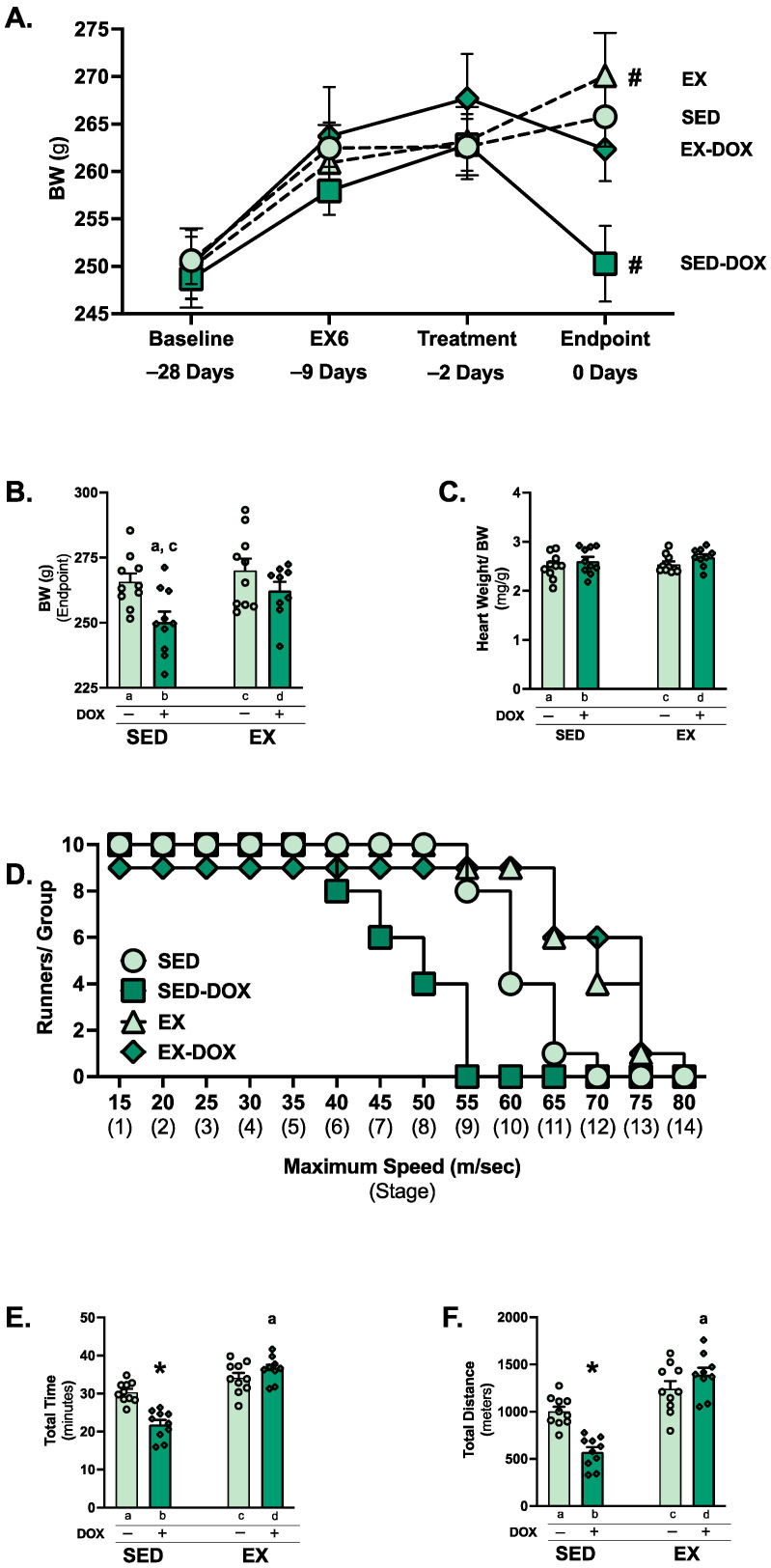
Animal characteristics and exercise tolerance test: body weight change over the experimental period (**A**), body weight at endpoint (**B**), and heart weight normalized to body weight at endpoint (**C**). Comparison of stages completed for the exercise tolerance test (ETT) (**D**), ETT total time ran (**E**), and ETT total distance ran (**F**). Values are reported as mean ± SEM. Significant differences are indicated by symbols and individual letters (a–d) located above relevant groups (*p* < 0.05). # = endpoint significantly different versus treatment (*p* < 0.05). * = significantly different versus all groups (*p* < 0.05). a = significantly different versus SED (*p* < 0.05). c = significantly different versus EX (*p* < 0.05).

Body weight loss and diminished physical capacity are typical consequences of cancer treatment and can be associated with poor long-term outcomes [28]. We observed that two weeks of moderate-intensity (~70% VO_2Max_) exercise preconditioning limited body weight loss in DOX-treated animals (Figure 1A). Body weight gain was observed for all groups from baseline (−28 days) to treatment (−2 days). Following saline treatment, exercise preconditioned rats’ (EX) body weight significantly increased, with no change in the sedentary (SED) rats. DOX treatment of sedentary rats (SED-DOX) resulted in significant body weight loss, while body weight was not different in rats that were exercise trained prior to DOX treatment (EX-DOX). Between the groups, body weight differences at endpoint demonstrated a significant reduction in the SED-DOX group compared to SED and EX (Figure 1B). No difference existed in heart weight/body weight (Figure 1C).

An exercise tolerance test (ETT) was performed to determine the effects of treadmill preconditioning on aerobic exercise capacity in DOX-treated rats. Our results demonstrate the distinct effects of both exercise and treatment, as SED-DOX rats ran for less total time and distance compared to all other groups (Figure 1D–F). In addition, EX-DOX rats ran a significantly greater amount of time and distance compared to the SED rats.

### 2.2. DOX Impairs Cardiac Function

**Figure 2:**
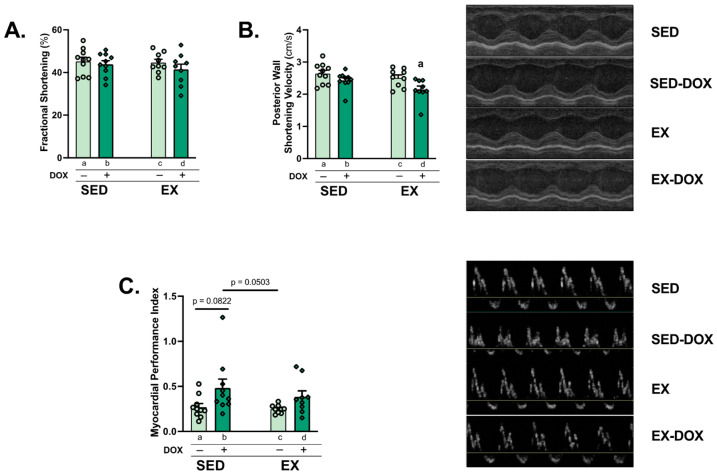
Cardiac performance: M-Mode and Doppler echocardiography were performed to evaluate fractional shortening (**A**), posterior wall shortening velocity (**B**), and myocardial performance index (**C**). Representative images are shown to the right of the graphs. Values are reported as mean ± SEM. Significant differences are indicated by individual letters (a–d) located above relevant groups (*p* < 0.05). a = significantly different versus SED (*p* < 0.05).

Differences in cardiac function between groups was determined by echocardiography. Analysis of left ventricular (LV) systolic function via fractional shortening showed no differences between groups. Posterior wall shortening velocity was significantly reduced in the EX-DOX group compared to SED (Figure 2A,B). The myocardial performance index was calculated as a marker of LV diastolic dysfunction. Comparison of SED-DOX against SED (*p* = 0.0822) and EX (*p* = 0.0503) approached significance (Figure 2C). No differences existed in posterior or anterior wall thickness during diastole or systole (Table 1). Heart rate was reduced in both DOX-treated groups compared to the saline controls, which was likely an effect of anesthesia.

### 2.3. Exercise Does Not Alter DOX or Doxorubicinol Levels

**Figure 3:**
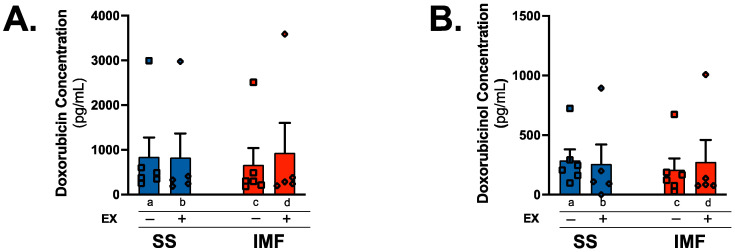
Doxorubicin & doxorubicinol quantification: concentration of doxorubicin (**A**) and doxorubicinol (**B**) within SS and IMF subfractions was evaluated with high performance liquid chromatography/electrospray ionization tandem mass spectrometry (HPLC-ESI-MS) and normalized to protein content of 10 µg per sample (n = 5–6/group). Values are reported as mean ± SEM. Significant differences are indicated by individual letters (a–d) located above relevant groups (*p* < 0.05).

DOX accumulates in the heart following administration, and increased mitochondrial levels of DOX are associated with cardiac and mitochondrial dysfunction [26,29]. Comparison of DOX concentration between groups showed no effect of exercise preconditioning and no differences between mitochondrial subfractions (Figure 3A). In addition, mitochondrial levels of the highly reactive DOX metabolite doxorubicinol (DOXol) were not different between groups or subfractions (Figure 3B).

### 2.4. DOX and Exercise Increase Endogenous Antioxidant Expression in SS Mitochondria

**Figure 4:**
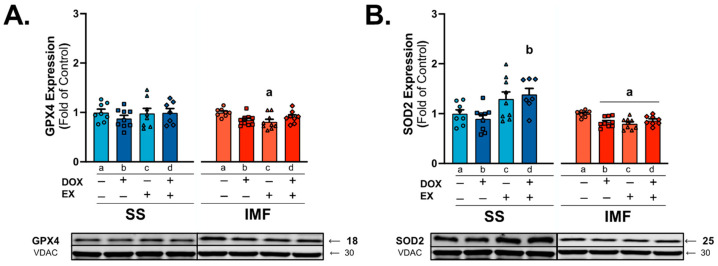
Endogenous mitochondrial antioxidants: western blot was performed for expression of glutathione peroxidase 4 (GPX4; (**A**)) and superoxide dismutase 2 (SOD2; (**B**)). Values are reported as mean ± SEM. Representative western blot images are shown below the graphs. Significant differences are indicated by individual letters (a–d) located above relevant groups (*p* < 0.05). a = significantly different versus SED (*p* < 0.05). b = significantly different versus SED-DOX (*p* < 0.05).

Oxidative damage to mitochondria is a primary cause of DOX cardiotoxicity, with increased antioxidant expression shown to prevent mitochondrial redox disturbances [14,30]. Analysis of protein expression of the mitochondria-localized antioxidant glutathione peroxidase 4 (GXP4) showed a reduction in GPX4 in EX rats compared to SED in the IMF fraction (Figure 4A). Evaluation of SOD2 expression showed a significant increase in SOD2 protein levels in EX-DOX rats compared to DOX in the SS subfraction. In contrast, SOD2 was reduced in all groups compared to SED for the IMF subfraction (Figure 4B).

### 2.5. Mitochondrial Iron Levels and Signaling in SS Mitochondria Are Affected by DOX Treatment and Exercise

**Figure 5:**
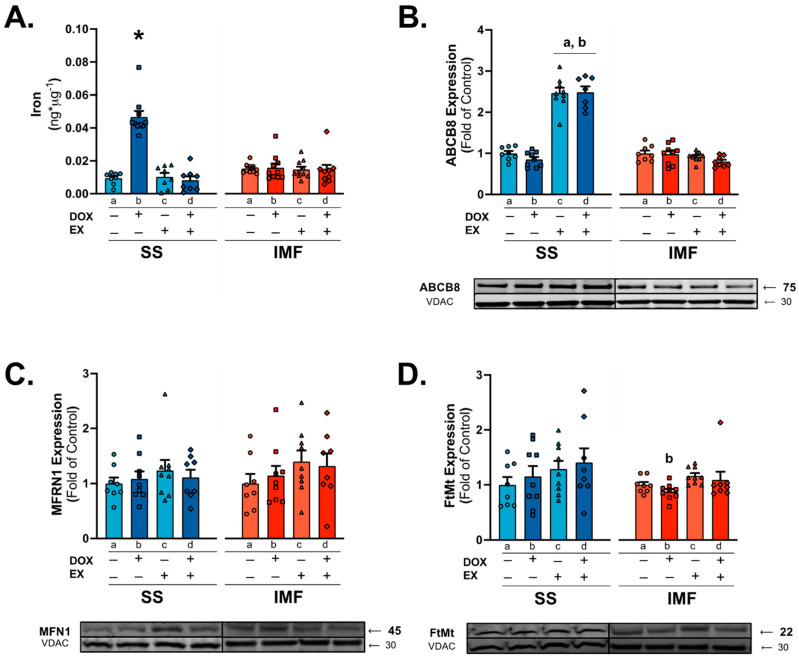
Mitochondrial iron quantification & signaling: concentration of iron (**A**) in SS and IMF mitochondria. Western blots were performed for protein expression of ATP-binding cassette subfamily B member 8 (ABCB8; (**B**)), mitoferrin-1 (MFRN1; (**C**)), and mitochondrial ferritin (FtMt; (**D**)). Values are reported as mean ± SEM. Significant differences are indicated by symbols and individual letters (a–d) located above relevant groups (*p* < 0.05). * = significantly different versus all groups (*p* < 0.05). a = significantly different versus SED (*p* < 0.05). b = significantly different versus SED-DOX (*p* < 0.05).

Reactions with iron can perpetuate mitochondrial ROS production through one- and two-electron reductions and/or oxidation of quinone and semi-quinone DOX modalities [31]. Quantification of mitochondrial iron levels revealed a significant increase in SS mitochondrial iron levels compared to all groups. No differences existed between groups for the IMF mitochondria (Figure 5A).

Evaluation of the mitochondria-localized iron exporter ATP-binding cassette subfamily B member 8 (ABCB8) showed a significant increase in protein expression in the EX and EX-DOX groups compared to SED and SED-DOX for the SS fraction. No differences existed between groups in the IMF mitochondria (Figure 5B). In addition, no differences existed between groups for the expression of mitochondrial iron importer mitoferrin-1 (MFRN1) or the mitochondrial iron storage protein mitochondrial ferritin (FtMt) (Figure 5C,D).

### 2.6. IMF Mitochondria Exhibit Increased Respiration Compared to SS Mitochondria

**Figure 6:**
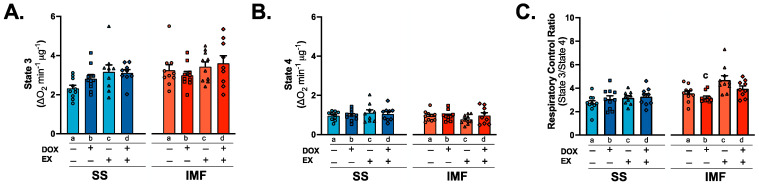
Mitochondrial respiration: state 3 (**A**), state 4 (**B**) and respiratory control ratio (**C**) for SS and IMF mitochondria. Values are reported as mean ± SEM. Significant differences are indicated by individual letters (a–d) located above relevant groups (*p* < 0.05). c = significantly different versus EX (*p* < 0.05).

**Figure 7:**
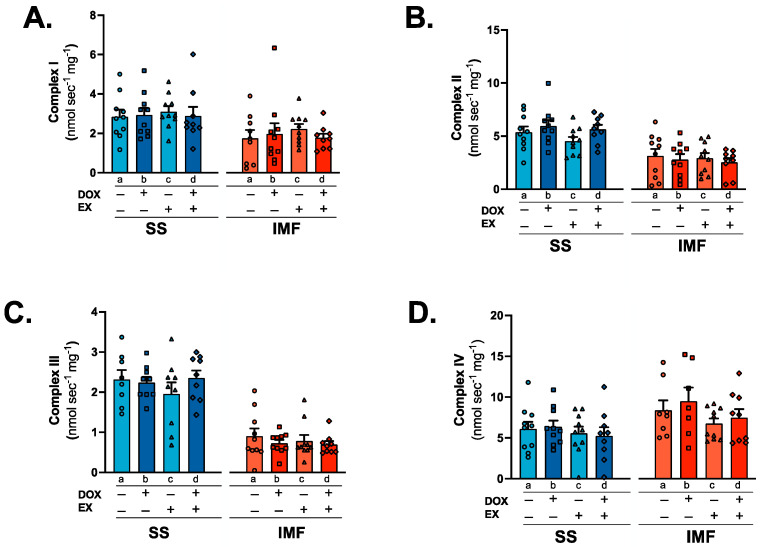
Electron transport chain complex performance: individual enzyme activity for SS and IMF complex I (NADH: ubiquinone oxidoreductase; (**A**)), complex II (succinate dehydrogenase; (**B**)), complex III (cytochrome c oxidoreductase; (**C**)), and complex IV (cytochrome c oxidase; (**D**)). Values are reported as mean ± SEM. Significant differences are indicated by individual letters (a–d) located above relevant groups (*p* < 0.05).

Complex I substrates (malate and pyruvate) were used to determine ADP-stimulated maximal oxygen consumption (state 3), uncoupled respiration (state 4) and the respiratory control ratio (state 3/state 4). No differences exist between groups for state 3 or state 4 respiration (Figure 6A,B). Calculation of the respiratory control ratio revealed a significant reduction in oxygen consumption in SED-DOX rats compared to EX in the IMF fraction (Figure 6C). To further clarify whether discrete effects exist between DOX and exercise on the individual mitochondrial complexes in the SS and IMF subpopulations, we employed specific enzymatic assays for the activity of mitochondrial complexes I-IV. These data show no differences between groups (Figure 7A–D).

**Figure 8:**
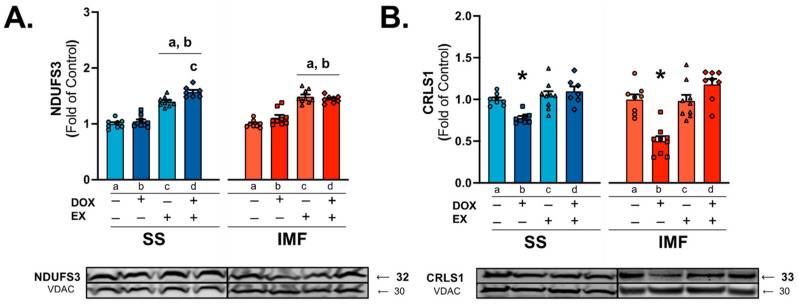
Mitochondrial complex I-associated protein expression: NADH: ubiquinone oxidoreductase core subunit S3 (NDUFS3) (**A**) and cardiolipin synthase 1 (CRLS1) (**B**) protein expression in mitochondrial subfractions. Values are reported as mean ± SEM. Representative western blot images are shown below the graph. Significant differences are indicated by symbols and individual letters (a–d) located above relevant groups (*p* < 0.05). * = significantly different versus all groups (*p* < 0.05). a = significantly different versus SED (*p* < 0.05). b = significantly different versus SED-DOX (*p* < 0.05). c = significantly different versus EX (*p* < 0.05).

Assessment of the core complex I subunit NADH: ubiquinone oxidoreductase (Complex I) core subunit S3 (NDUFS3) showed a significant effect of exercise in both the SS and IMF mitochondria, with EX and EX-DOX showing greater protein expression compared to both the SED and SED-DOX groups (Figure 8A). Protein expression of cardiolipin synthase 1 (CRLS1) also revealed significant difference between groups, with protein levels significantly reduced in the SED-DOX group compared to all other groups (Figure 8B). This result was consistent between both the SS and IMF mitochondria.

## 3. Discussion

DOX is one of the most widely used antineoplastic agents. However, the incidence of cardiac dysfunction in cancer patients receiving DOX treatment severely limits its use [6,7,8,9]. Efforts to elucidate the mechanism(s) of DOX cardiotoxicity have primarily focused on the role of mitochondrial dysfunction, due to its localization to cardiolipin on the inner mitochondrial membrane and subsequent disruption of the respiratory chain complexes and mitochondrial membrane [7,9,14]. Contrary to this, exercise training can upregulate antioxidant defenses, reduce electron transport chain leakage, and stabilize the outer mitochondrial membrane [32,33]. Results from the current study expand on these previous findings and demonstrate that exercise preconditioning exerts distinct adaptations to SS and IMF mitochondria which reduce their susceptibility to DOX.

### 3.1. Mitochondria Localization of DOX

Cardiac mitochondrial dysfunction is a multi-factorial consequence of DOX exposure [7,9,14]. Following cardiomyocyte entry, the mitochondria are a primary target of DOX due to its cationic and hydrophilic properties and affinity for cardiolipin. Indeed, experiments using both cell culture and cardiac tissue confirm the preferential accumulation of DOX within the mitochondrial fraction compared to the cytoplasm [7,34,35]. Limited evidence also suggests greater accumulation within the SS mitochondria compared to the IMF [26], and that exercise preconditioning can reduce the mitochondrial concentration of DOX [29]. However, the current study was not able to distinguish differences between the subpopulations or treatment groups. The cause for these discrepancies is unclear but could result from the sample size and different methodologies used. Interestingly, levels of DOXol, the principal metabolite of DOX, also did not differ between groups. While the precise understanding of these findings is unclear, it may suggest differences in the rate of metabolic elimination of DOX. Data suggest that DOXol itself is highly toxic and can promote cardiac dysfunction [36]. Therefore, further investigation is needed to determine the precise effects of exercise training on DOX localization and metabolism.

### 3.2. Mitochondrial Antioxidant Enzymes

Endogenous antioxidant expression is altered by both DOX and exercise and may play an important role in reducing oxidative damage caused by excessive ROS production [37]. Within the mitochondria, SOD2 (MnSOD) expression has the potential to play a major role in free radical detoxification and preservation of cardiac function [38]. Specifically, transgenic overexpression of SOD2 in the heart of DOX-treated mice improved cardiac and mitochondrial morphology [39], and the exercise-induced increase in SOD2 expression is associated with a cardioprotective phenotype following ischemia reperfusion injury [38,40]. While the precise contribution of SOD2 has not been determined regarding exercise protection and DOX, our results and others show a positive relationship between cardiac function and SOD2 expression [33,41]. However, the benefit of SOD2 may be isolated to the SS subfraction, as IMF mitochondria did not upregulate SOD2 in response to exercise preconditioning [27].

### 3.3. Mitochondrial Iron Handling

Mitochondrial iron overload has been observed in the hearts of patients suffering from DOX cardiomyopathy and is heavily implicated in mitochondrial dysfunction due to the disruption of electron transport chain cofactor synthesis and excessive ROS generation [42]. Perturbation of mitochondrial iron homeostasis results from inactivation of iron regulatory proteins 1 and 2 by DOX and DOXol, which results in increased cellular uptake of iron through the transferrin receptor [31]. Inside the cell, mitochondrial iron import is regulated by MFRN; it is stored in the mitochondria by FtMt and efflux is primarily regulated by the ABCB8 transporter [31]. Following DOX treatment, MFRN and ABCB8 levels have been shown to decrease and FtMt to increase [42]. Comparison of iron levels between SS and IMF mitochondria show a preferential increase in iron within the SS fraction that is attenuated with exercise. This change in mitochondrial iron accumulation appears to be related to exercise-induced increases in ABCB8 protein expression.

### 3.4. Mitochondrial Respiration and Complex Activity

At the inner mitochondrial membrane, DOX and its metabolites can severely impair complex electron transport function and enzyme activity [13,43,44]. While it is established that DOX can impair the mitochondrial respiratory control ratio, substrate-specific differences exist between the subfractions. Utilization of glutamate or palmitoyl-CoA/carnitine revealed a reduction in oxygen consumption in both SS and IMF mitochondria, whereas pyruvate/malate-driven oxygen consumption is only reduced in the IMF mitochondria compared to the SS [25,26]. Exercise appears to preferentially increase pyruvate/malate-stimulated respiratory control ratio in the IMF fraction. This improvement may be related to changes in the electron transport system, as exercise also increased the concentration of the complex I subunit NDUFS3.

Cardiolipin is another key determinant of mitochondrial bioenergetic efficiency. It is synthesized by cardiolipin synthase (CRLS), and the maintenance of both cardiolipin and CRLS content is required to maintain mitochondrial function [45]. Decreased cardiac mitochondrial cardiolipin and CRLS1 content occurs as a consequence of DOX treatment, and alterations to cardiolipin content are associated with heart failure [7,46]. However, targeting of CRLS may be an effective therapeutic, as increased expression of CRLS, independent of an increase in total cardiolipin content, was sufficient to preserve mitochondrial bioenergetic efficiency and metabolic capacity in diabetic myocardium [47]. Results from this study reveal a role for CRLS in the development of DOX-induced mitochondrial toxicity, as well as a beneficial effect of exercise of upregulating its expression in both the SS and IMF mitochondria.

## 4. Materials and Methods

### 4.1. Experimental Animals

Study approval was obtained from the University of Florida Institutional Animal Care and Use Committee (IACUC approval #202011110) and experiments were performed in accordance with the National Institutes of Health Guide for the Care and Use of Laboratory Animals [48]. Forty female Sprague Dawley rats (4–6 months old) were used in these experiments (Charles River Laboratories, Wilmington, MA, USA). Female rats were chosen because of the widespread use of DOX in the treatment of breast and gynecological cancers [49,50]. Upon arrival, rats were acclimated, weighed and randomly allocated into experimental groups for even weight distribution. Rats were housed in pairs with food and water provided ad libitum on a 12:12 light–dark cycle.

### 4.2. Study Design

Rats were divided into 4 groups (n = 10/group) based on sedentary/exercise training or saline/DOX treatment: (1) sedentary-saline (SED), (2) sedentary-DOX (SED-DOX), (3) exercise-saline (EX), and (4) exercise-DOX (EX-DOX). Exercise trained rats were habituated to treadmill running for 5 days at a speed of 30 m/min and 0% grade for 10, 20, 30, 40, and 50 min on days 1–5. Following 2 days of rest, animals underwent 10 days of exercise training with 2 days of rest after day 5. Exercise bouts were performed for 60 min per day at 30 m/min and 0% grade. Sedentary animals were exposed to a still treadmill on 10 individual occasions. 5 days before treatment, sedentary groups were progressively habituated: day 1 (10 min @ 10 m/min), day 2 (12 min @ 10 m/min), day 3 (10 min @ 12 m/min), day 4 (12 min @ 12 m/min), day 5 (4 min @ 10, 12 and 15 m/min each). Twenty-four hours after the final exercise bout or habituation, rats received either saline or DOX treatment. DOX groups received Doxorubicin HCl (McKesson, Irving, TX, USA), administered I.P. at 20 mg/kg [29,51]. Saline groups received an equal volume of saline. Forty-eight hours following treatment, animals were anesthetized with 2–4% inhaled isoflurane via nose cone (SomnoSuite, Kent Scientific, Torrington, CT, USA) to undergo echocardiography followed by euthanasia. Hearts were excised, cleared of extraneous debris, and lightly blotted before obtaining wet weight and beginning mitochondrial isolation. One animal in the EX-DOX group was excluded from the study due to cardiac abnormalities independent of exercise training or DOX treatment.

### 4.3. Exercise Tolerance Test

Prior to euthanasia, all rats were administered an exercise tolerance test (ETT) by a blinded researcher based on a validated protocol for exhaustion [52]. The ETT began at 10 m/min and increased 5 m/min every 3 min until animals were no longer able to run. Exhaustion was confirmed immediately following the completion of the ETT [53,54,55].

### 4.4. Echocardiography

Transthoracic echocardiography (LogiQe NextGen, SOUND Technologies, Carlsbad, CA, USA) was performed under inhaled isoflurane as described previously [51,56]. The LV systolic function was determined via assessment of fractional shortening, posterior wall shortening velocity, and wall thickness using M-mode echocardiography. Doppler imaging was used to assess the myocardial performance index as a marker of LV diastolic dysfunction. Analysis of echocardiographic outcomes was performed in Fiji (Fiji Is Just ImageJ, NIH, Bethesda, MD, USA). Image acquisition and analysis were completed by researchers blinded to the experimental groups.

### 4.5. Mitochondrial Isolation

The entire heart was used for mitochondrial isolation using a previously described protocol [26]. Hearts were minced for 5 min in 10× mg-volume stable buffer [solution 1: 100 mM KCl (Sigma, St. Louis, MO, USA, P4504), 50 mM MOPS (Sigma M1254), 5 mM MgSO_4_ (Sigma M2643), 1 mM EGTA (Sigma E4378), 1 mM ATP (Sigma M2643), 0.2% BSA (Sigma A6003), pH 7.4 at 4 °C]. Contents were briefly processed with a mechanical homogenizer, followed by a PFTE Dounce homogenizer and brief centrifugation (500× *g*, 10 min, 4 °C) to separate the SS (supernatant) from IMF (pellet) mitochondria. The IMF subfraction was processed through mechanical homogenization again and underwent trypsin digestion [1× mg-volume (5 mg trypsin/g of tissue; Sigma T1426) prepared in solution 1]. Digestion was terminated with the addition of 10× mg-volume of solution 1. SS and IMF mitochondria underwent several washing steps with solution 1 and centrifugation (3500× *g*, 10 min, 4 °C), with a final pelleting spin in solution 2 (solution 1 without BSA). The mitochondrial pellet was resuspended in 250 µL of resuspension buffer [220 mM mannitol (Sigma M9467), 70 mM Sucrose (Sigma S9378), 10 mM Tris-HCl (VWR 0278, Radnor, PA, USA) and 1 mM EGTA (Sigma E4378), pH 7.4 at 4 °C], using separate PFTE Dounce homogenizers for each fraction.

### 4.6. Doxorubicin and Doxorubicinol Concentration

The concentration of DOX and DOXol in mitochondrial subfractions isolated from DOX-treated hearts were evaluated via high performance liquid chromatography/electrospray ionization tandem mass spectrometry (HPLC-ESI-MS/MS) at the University of Florida Mass Spectrometry Core. Samples were quantified utilizing the Bradford method, normalized for protein content, blinded, and coded before transferring to the core lab for analysis. The protein samples were denatured by methanol (Thermo Fisher Scientific, Waltham, MA, USA) and sonicated for 10 min. Samples were then centrifuged at room temperature, 10,000× *g* for 10 min. Following the first centrifugation, the supernatants were collected, dried, and concentrated via SpeedVac (Thermo Fisher Scientific), followed by reconstitution in methanol.

Aliquots of 10 µL were applied in reverse phase HPLC-ESI-MS/MS (Thermo Fisher Scientific, Ultimate 3000), using a C8 column (Thermo Fischer Scientific, Hypurity C8, 5 µm × 2.1 × 100 mm). The elution was carried out by applying a linear gradient from 95% of solvent A (0.1% of formic acid in water) and 5% of solvent B (0.1% of formic acid in methanol) to 100% of solvent over a period of 90 min (flow rate 0.2 mL/min). Mass spectrometry was obtained using LTQ XL linear quadrupole ion trap mass spectrometer with ESI (Thermo Fisher Scientific) operating with XCALIBUR 2.2 SP1.48. The molecular weight (MW) of doxorubicinol (545) was monitored via *m*/*z* 546–*m*/*z* 399, and the MW of Doxorubicin (543) was monitored via *m*/*z* 544–*m*/*z* 397. Doxorubicin and doxorubicinol concentration are expressed as pg mL^−1^.

### 4.7. Mitochondrial Iron

Assessment of iron quantity (ng) utilizes a commercially available colorimetric assay kit (Abcam, Waltham, MA, USA, ab83366) and was performed per manufacturer’s instructions in mitochondrial subfractions. Isolated subfractions were diluted to 100 µg of protein per reaction in iron assay buffer (provided in kit), performed in duplicate (n = 8–10/group), and averaged. Standard curves were performed, with each assay ranging from 0–10 ng of iron.

### 4.8. Mitochondrial Respiration

Oxygen consumption was measured polarographically in water-jacketed respiration chambers maintained at 37 °C (Oxygraph, Hanstech Instruments, King’s Lynn, UK) as previously detailed [56]. Following daily calibration with Buffer Z [50 mM K-MES (Sigma M0895), 30 mM KCl (Sigma P4504), 10 mM K_2_HPO_4_ (Fisher, Hampton, NH, USA, P290), 1 mM EGTA (Sigma E4378), 5 mM MgCl_2_-6H_2_O (Sigma M2670), 0.005 mM Glutamate (Sigma G8415), 0.002 mM Malate (Sigma M6773), and 0.05% BSA (Sigma A6003), pH to 7.1 at 4 °C] and Na_2_SO_4_, 10 µL of isolated SS or IMF mitochondria was independently resuspended in 965 µL of buffer Z, containing 20 mM creatine (Sigma C0708) warmed to 37 °C within the oxygraph chamber. Mitochondria were allowed to equilibrate before the addition of 10 µL malate (272.21 mM; Sigma M7397) and 10 µL pyruvate (500 mM; Sigma P5280) followed by the addition of 5 µL of ADP (48.09 mM; MP Biomedicals, Irvine, CA, USA, 150259) to determine state 3 and state 4 respiration. Respiratory control ratio (RCR) was designated as state 3 respiration divided by state 4 respiration. Values were normalized post hoc to protein content by the Bradford method.

### 4.9. Mitochondrial OXPHOS Complex Activity

Samples were assessed for protein concentration and diluted to 0.8 µg/µL in a hypotonic buffer (2.5 mM K_2_HPO_4_ (Sigma P3786) and 5.3 mM MgCl_2_ (Sigma M8266) in DI water, pH 7.2). Samples underwent three freeze–thaw cycles in the hypotonic buffer and were stored at −80 °C until time of assay, where they were re-warmed at 37 °C for ~60 s. Samples were run in duplicate and averaged on a 96-well plate in a SpectraMax M5 plate reader (Molecular Devices, San Jose, CA, USA) over a linear range of 60 s at 37 °C. Assays and calculations reflect the protocols detailed in several manuscripts [57,58,59].

Enzyme Activity (nmol min^−1^ mg^−1^) = (∆Absorbance/min * 1000)/[(extinction coefficient * volume of sample use in mL) * sample protein concentration in mg/mL)] (1)

Analysis of enzyme activity utilizes Equation (1):

Complex I analysis utilizes assay buffer (5 mM Tris-HCl, 0.5% BSA, 0.024 mM KCN (Sigma 60178) and 0.0004 mM Antamycin A (AMA; Sigma A8674), pH 7.2) reacting with 1 mM 2,6-Dichloroindophenol Sodium Salt Hydrate (DCPIP; Sigma D1878), 1 mM ß-Nicotinamide adenine dinucleotide hydrate (NADH; Sigma N4505), and 40 mM Oxidized Decycloubiquinone (DCU; Sigma D7911), with Rotenone (0.025 mM; Sigma R8875) in inhibitor wells. 10 µg of isolated mitochondria was used per reaction. Samples were run in duplicate for inhibited and non-inhibited wells and read at 600 nm, with an extinction coefficient of 19,100 M^−1^cm^−1^.

Complex II utilizes an assay buffer (10 mM KH_2_PO4, 1% BSA, 2 mM EDTA (Sigma 34103), pH 7.8) reacting with 0.004 mM Rotenone, 0.2 mM ATP, 10 mM Succinate and 0.08 mM DCPIP, with 10 mM Malonate (Sigma M1296) in inhibitor wells. 5 µg of isolated mitochondria was used per reaction. Samples were run in duplicate for inhibited and non-inhibited wells and read at 600 nm, with an extinction coefficient of 19,100 M^−1^cm^−1^.

Complex III utilizes the same assay buffer as complex II to react with 0.15 mM reduced DCU, 0.2 mM ATP (Sigma A9062), 0.24 mM KCN and 0.11 mM cytochrome c (Sigma C2506), with 0.1 mM Myxothiazol (Sigma T5580) in inhibitor wells. 20 µg of isolated mitochondria was used per reaction. Samples were run in duplicate for inhibited and non-inhibited wells and read at 550 nm, with an extinction coefficient of 18,500 M^−1^cm^−1^.

Complex IV utilizes an assay buffer (10 mM KH_2_PO4, 1% BSA, 250 mM Sucrose, pH 7.8) to react with 0.1 mM reduced cytochrome c, 2.5 mM n-dodecyl β-D-Maltoside (Sigma D46410) and 0.005 mM AMA, with 24 mM KCN in inhibitor wells. Reduction of cytochrome c with sodium dithionite was confirmed via color change and readings at 550 nm vs. 565 nm with a ratio of 6.33 as previously indicated for proper reduction [57]. 3 µg of isolated mitochondria was used per reaction. Samples were run in duplicate for inhibited and non-inhibited wells and read at 550 nm, with an extinction coefficient of 18,500 M^−1^cm^−1^.

### 4.10. Western Blot Analysis

Isolated mitochondria were taken through three freeze (−80 °C)–thaw (37 °C) cycles, followed by analysis of protein content via the Bradford method. Approximately 30–45 µg of protein were separated on 4–20% precast gels (Bio-Rad, Hercules, CA, USA) and transferred to nitrocellulose membranes, followed by blocking in 5% non-fat milk, washing in PBST, and a minimum of one overnight incubation at 4 °C with primary antibodies directed against GPX4 (ab125066, 1:1000); MFRN1 (Mitoferrin1, ab56134, 1:1000); SOD2 (ab68155, 1:1000); VDAC (ab14734, 1:1000) from Abcam; ABCB8 (TA322438, 1:1000) from Origene (Rockville, MD, USA); FtMt (PA530906, 1:500) from Invitrogen (Waltham, MA, USA); CRLS1 (14845-1-AP); and 1:1000 NDUFS3 (15066-1-AP, 1:1000) from Proteintech (Rosemont, IL, USA) diluted 1:1 in Odyssey blocking buffer (LI-COR Biosciences, Lincoln, NE, USA) and PBS. Membranes were exposed to rabbit or mouse AlexaFluor 680 IgG or 800 IgG (LI-COR) secondary antibodies. Imaging and analysis were performed using the Odyssey CLx imaging system and Image Studio software (LI-COR).

### 4.11. Statistical Analysis

Data are presented as mean ± SEM. The Shapiro–Wilk test was used to test for normal distribution. Data with normal distribution were compared by one-way analysis of variance (ANOVA) to determine whether differences existed between groups, with a Tukey test performed post hoc when the ANOVA was significant. Nonparametric data was analyzed using the Kruskal–Wallis test, followed by Dunn’s post hoc test when appropriate. Significance was established at *p* < 0.05.

## 5. Conclusions

These data confirm the importance of mitochondrial function in the maintenance of cardiorespiratory capacity following preconditioning exercise and DOX administration. In addition, they provide new and important information regarding the SS and IMF mitochondrial subpopulations in response to DOX and exercise. Our data reveal that, compared to IMF mitochondria, the SS subfraction are greatly affected by the destabilization of iron homeostasis, and that exercise confers resistance to mitochondrial iron overload primarily through upregulation of antioxidant defenses and iron efflux transporter expression. We also show that complex I efficiency is reduced in IMF compared to SS mitochondria. Importantly, this work identifies CRLS as a novel therapeutic target induced by exercise preconditioning, and future work is needed to determine its potential contribution to exercising cardioprotection against DOX toxicity.

## Figures and Tables

**Table 1:** Cardiac function. Posterior (P) and anterior (A) wall thickness (WT; millimeters (mm)) during diastole (d) and systole (s) and heart rate (HR; beats per minute, bpm). Values are reported as mean ± SEM with significance set at *p* < 0.05. Significant differences are indicated next to relevant measures, ^ = significantly different versus EX (*p* < 0.05).

	SED	SED-DOX	EX	EX-DOX
PWTd (mm)	1.79 ± 0.10	1.80 ± 0.08	1.72 ± 0.03	2.02 ± 0.14
PWTs (mm)	2.79 ± 0.16	2.72 ± 0.10	2.62 ± 0.07	2.88 ± 0.13
AWTd (mm)	1.70 ± 0.11	1.71 ± 0.08	1.78 ± 0.12	1.78 ± 0.09
AWTs (mm)	2.75 ± 0.17	2.67 ± 0.10	2.75 ± 0.20	2.69 ± 0.14
HR (bpm)	363.9 ± 7.01	347.0 ± 4.80 ^^^	380.8 ± 15.39	336.2 ± 5.86 ^^^

## Data Availability

The authors confirm that the data supporting the findings of this study are available within the article and/or are available upon reasonable request, further questions can be directed to the corresponding author.
